# Comparison of Body Scanner and Manual Anthropometric Measurements of Body Shape: A Systematic Review

**DOI:** 10.3390/ijerph18126213

**Published:** 2021-06-08

**Authors:** Lorena Rumbo-Rodríguez, Miriam Sánchez-SanSegundo, Rosario Ferrer-Cascales, Nahuel García-D’Urso, Jose A. Hurtado-Sánchez, Ana Zaragoza-Martí

**Affiliations:** 1 Department of Nursing, University of Alicante, 03690 Alicante, Spain; lrr51@gcloud.ua.es (L.R.-R.); ja.hurtado@ua.es (J.A.H.-S.); ana.zaragoza@ua.es (A.Z.-M.); 2 Department of Health Psychology, University of Alicante, 03690 Alicante, Spain; rosario.ferrer@ua.es; 3 Department of Computer Technology, University of Alicante, 03690 Alicante, Spain; nahuel.garcia@ua.es; 4 Alicante Institute for Health and Biomedical Research (ISABIAL-FISABIO Foundation), 03010 Alicante, Spain

**Keywords:** whole-body imaging, body scanner, anthropometry, waist circumference, reliability, validity

## Abstract

Anthropometrics are a set of direct quantitative measurements of the human body’s external dimensions, which can be used as indirect measures of body composition. Due to a number of limitations of conventional manual techniques for the collection of body measurements, advanced systems using three-dimensional (3D) scanners are currently being employed, despite being a relatively new technique. A systematic review was carried out using Pubmed, Medline and the Cochrane Library to assess whether 3D scanners offer reproducible, reliable and accurate data with respect to anthropometrics. Although significant differences were found, 3D measurements correlated strongly with measurements made by conventional anthropometry, dual-energy X-ray absorptiometry (DXA) and air displacement plethysmography (ADP), among others. In most studies (61.1%), 3D scanners were more accurate than these other techniques; in fact, these scanners presented excellent accuracy or reliability. 3D scanners allow automated, quick and easy measurements of different body tissues. Moreover, they seem to provide reproducible, reliable and accurate data that correlate well with the other techniques used.

## 1. Introduction

Anthropometrics are a set of direct quantitative measurements of the human body’s external dimensions, which can be used as indirect measures of body composition. [1]. The most important elements of anthropometry include height, weight, body mass index (BMI), body circumferences (waist, hip and limbs) and skinfold thickness [1]. These measures are of great interest to dietitians-nutritionists, health professionals and sports professionals because of their clinical utility. On the one hand, these measurements represent diagnostic criteria for obesity, which significantly increases the risk of cardiovascular diseases and diabetes mellitus, among other disease. Anthropometry is important not only in public health but also in clinical and community nutrition for the design of nutritional strategies and the monitoring of therapeutic interventions. On the other hand, these measurements can also be used as a basis for measuring physical fitness and fitness progress [2].

Currently, there are different techniques for evaluating body composition, ranging from simple indirect measurements to more sophisticated direct volumetric measurements. Conventional manual methods of collecting body measurements using anthropometers, calipers and measuring tapes are simple and inexpensive. However, they have some limitations such as (a) long application time, (b) the need for careful calibration of equipment and trained observers, (c) changes in the patient’s body posture, (d) variations in tape pressure during measurement, and (e) the identification of reference points, which can be more of a problem in people with higher body fat [3,4,5].

Currently, in an attempt to overcome these limitations, despite being a basically new technique in the health area, advanced anthropometric measurement systems utilizing three-dimensional scanners are being used [4,5]. For several years, the technology has been used to measure the 3D shape of an object but is now able to accurately and precisely measure shapes of the human body [6]. 3D and 4D body scanners have proven to be efficient and versatile, while being less time consuming and invasive than conventional anthropometry and other whole-body imaging methods, such as computed tomography (CT) and dual-energy X-ray absorptiometry (DXA) [6,7]. However, these devices can also lead to errors; therefore, they should be evaluated and validated before use because the most important evaluation criterion of any new measurement technology is its ability to obtain reliable, precise, and accurate data [4].

Thus, the aim of this study was to collect the existing information in the literature regarding the validation of different three-dimensional scanners for taking anthropometrics and their usefulness in clinical practice to determine whether this type of system provides reproducible, reliable and accurate data. We hypothesize that the use of 3/4D body volume and composition measurement technologies will improve measurement accuracy over manual techniques.

## 2. Materials and Methods

This study employs a systematic review methodology, based on the PRISMA statement (not registered).

### 2.1. Data Sources

A systematic search was conducted in Pubmed, Medline and the Cochrane Library. Additional articles were identified from references in other articles.

### 2.2. Search Strategy

The search strategy aimed to identify published studies available in full text. A block search strategy was used, using medical subject headings (MeSH) descriptors and terms in titles or abstracts, as follows: “whole body imaging”, “body scanner”, “body scanning”, “3d scanner”, “3d images”, “three dimensional imaging”, “anthropometry”, “anthropometrics”, “anthropometric measures”, “waist circumference”, “hip circumference”, “waist circumference”, “reproducibility of results”, “validity”, “validation” and “reliability” joined by Boolean operators (AND, OR) as follows: (whole body imaging OR body scanner OR body scanning OR 3d images OR three dimensional imaging) AND (anthropometry OR anthropometrics OR anthropometric measures OR waist circumference OR hip circumference) AND (reproducibility OR validity OR validation OR reliability). The last search performed was on February 12, 2021, and no time restrictions were made regarding the year of publication. Table 1 shows the search strategy used in the Pubmed database.

### 2.3. Selection of Articles

The abstracts identified through the literature search were evaluated independently by two authors to determine if they met the inclusion criteria. The quality of each study was assessed independently by two authors using the Crombie criteria adapted by Petticrew and Roberts [8]. Disagreements were resolved by a third author.

A critical appraisal tool was used to assess quality and risk of bias in cross-sectional studies (AXIS) [9]. In general, it can be said that the quality of the cross-sectional studies included in this review was good (Table 2 and Table 3).

### 2.4. Inclusion and Exclusion Criteria

The inclusion criteria were (1) articles that were available in full text and written in English or Spanish, (2) articles in which the participants were 18 years of age or older, (3) articles that used 3D scanners and reference methods such as conventional anthropometry, DXA or plethysmography (ADP) and (4) using techniques such as electrical bioimpedance and hydrostatic weighing.

The exclusion criteria were (1) articles not related to the subject of the study or articles that were intervention protocols without results, (2) articles that were reviews or meta-analyses and (3) articles that evaluated only geometric shapes.

### 2.5. Extracted Data

Data extraction was performed by the lead author of the review, taking into consideration year of publication (1994–2020), objective of the study, sample size and age of the participants, measurement techniques, clothing and position during measurement, measurements taken, person in charge of taking the measurements, number of times measurements were taken, analyses performed, results obtained and conclusions.

### 2.6. Synthesis of Results

Once data extraction was completed, the results were grouped based on the measurement technique used for the evaluation of body measurements versus 3D scanners (1. 3D scanners and conventional anthropometry and 2. 3D scanners, dual-energy X-ray absorptiometry (DXA), plethysmography (ADP), bioelectrical impedance (BIA) and hydrostatic weighing) and it was thus observed to what extent 3D scanners provide reliable, precise and accurate data.

## 3. Results

**Figure 1:**
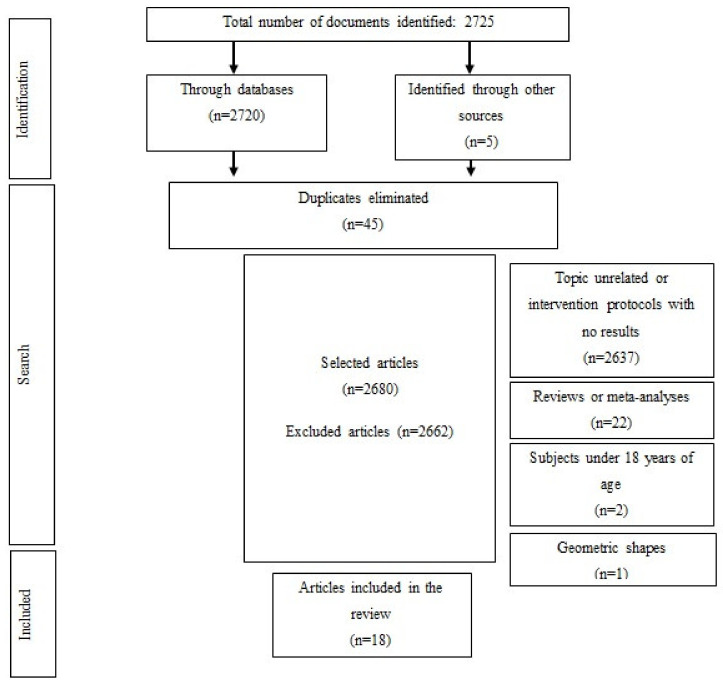
Selection of studies.

In total, 2725 studies were identified. After eliminating duplicates (n = 45), titles and abstracts were read, and a further 2662 articles were eliminated based on the exclusion criteria. Ultimately, 18 articles were included in this review (Figure 1).

### 3.1. Descriptive Data and Types of Studies

Table 4 shows the characteristics of the included articles. Of the participants, 51.22% were men, and the remaining 48.78% were women, with the mean age of the participants being approximately 32 years. Regarding the country of origin, half of the articles, i.e., 50.0%, were conducted in the United States (n = 9), two studies were conducted in Germany, and another two were conducted in the United Kingdom; one study was conducted in China, Canada, Mexico, the Netherlands and Switzerland.

Table 4 also identifies the design of the studies, showing that they were all cross-sectional studies.

Data related to the measurement process can be seen in the Supplementary Materials Table S1.

### 3.2. Validation of 3D Scanners for Taking Body Measurements

#### 3.2.1. 3D Scanners and Conventional Anthropometry

Six articles compared different types of 3D scanners and conventional anthropometry techniques (Table 5), demonstrating strong correlations between 3D scanner-based measurements and manual methods [12,13,15,21,22,24]. Ng et al. [13] found significant differences in measurement accuracy for waist circumference (1.75 cm) and hip circumference (3.17 cm). Measurements obtained through the 3D scanner were strongly associated with conventional anthropometry measurements (R^2^ = 0.95 and 0.92, respectively). Similarly, Ramos-Jiménez et al. [22] found that all 3D measurements were highly correlated with those obtained manually (R^2^ ≥ 0.75) but that significant differences existed for all of them (*p* < 0.01). Likewise, Koepke et al. [24] found significant differences between body composition measurement methods (*p* < 0.001), with higher accuracy rates based on the nature of the technologies. Both methods showed high correlations in the measurement of waist circumference (CCC > 0.94), chest circumference (0.781) and hip circumference (0.784) but not so for buttock circumference (0.258). Only in one study did three-dimensional and manual waist and hip measurements not differ significantly (*p* > 0.05), although both techniques showed a strong relationship (waist: r = 0.998 and hip: r = 0.989) [17].

Vonk & Daanen, [19], compared the measurement accuracy of two 3D volumetric body scanners with that of conventional anthropometry. The results using the SizeStream scanner demonstrated a high intraclass correlation coefficient with intervals between ICC < 0.80 and ICC > 0.90. In addition, strong correlations were observed for chest, waist and hip circumferences (R^2^ = 0.95; *p* < 0.001; R^2^ = 0.92; *p* < 0.001; R^2^ = 0.96; *p* < 0.001) despite the significant differences found for all of them (*p* < 0.001); using the scanner showed the greatest precision. These results were similar to those obtained with the Poikos scanner (ICC < 0.80). Regarding the comparison with the manual technique, for the Poikos scanner, no significant differences were found between the measurements, with the exception of waist circumference (*p* < 0.001). The scanner measurements were correlated to the manual measurements (R^2^ < 0.60).

In relation to the reliability and/or accuracy of the measurements, four articles reported that the 3D scanner was more accurate and/or reliable than conventional anthropometry. In particular, the interobserver variations (precision) for waist and hip circumferences were greater than the variability obtained for the 3D scanner (conventional anthropometry: waist: 3.9 cm and hip: 2.4 cm vs. 3D scanner: waist: 1.3 cm and hip: 0.8 cm) [15]. Koepke et al. [24], on the other hand, with the exception of hip circumference, found no significant differences for repeated 3D measurements, while significant differences were found for manual measurements. Furthermore, the precision of the manual measurements was higher than 2.50 cm, up to 8.19 cm, indicating greater disagreement. Other studies, however, have not found superior reliability in the use of scanning techniques. In particular, Bragança et al. [4] found that the 3D scanner was less reliable and accurate than conventional anthropometry because the 3D measurements presented higher standard errors of measurement for all measurements, with the exception of neck circumference. Moreover, both technical errors of measurement (TEM) and relative technical errors of measurement (%TEM) were better for the manual technique (Table 5).

#### 3.2.2. 3D Scanners, Dual-Energy X-ray Absorptiometry (DXA), Plethysmography (ADP), Bioelectrical Impedance (BIA) and Hydrostatic Weighing

Four of the six articles that compared different types of 3D scanners with a measurement technique other than conventional anthropometry found a high correlation between the accuracy of measurements taken when comparing between methods [10,11,13,20]. In the work by Adler et al. [10] and Bourgeois et al. [11], the body volume measured by 3D scanners correlated strongly with that measured by plethysmography (R^2^ = 0.99 in both studies). However, significant differences were observed between 3D and ADP body volume measurements. In the study by Adler et al., [10], the body volume measured by ADP was 72.2 L, and those measured by the 3D scanner were slightly higher by 1.1 L (*p* < 0.001), 1.0 L (*p* < 0.001) and 2.5 L (*p* < 0.001) in standard, relaxed and exhaled positions, respectively, whereas in the study by Bourgeois et al. [11], all three 3D scanners obtained lower values than that obtained by ADP (ADP: 76.4 L; KX-16: 73.0 L; Proscanner: 74.0 L; Styku: 67.4 L; *p* < 0.0001 for all). Similarly, Ng et al. [13] found that the total body volume measured by 3D scan was significantly lower than that obtained by ADP (-4.15 L) and that the regional volume estimated by 3D scan was lower than that estimated by DXA for the arm and leg but higher than that for the trunk (*p* < 0.001). However, there was a strong correlation between the total body volume and the regional volume measured by the 3D scanner and those measured by ADP and DXA (body volume Total: R^2^ = 0.99 and 0.97 respectively; and Body volume Regional: R^2^ = 0.73–0.97) [13]. Finally, Tinsley et al. [20] stated the existence of relatively poor validity for total and regional body volumes (Table 5).

Regarding the reliability and/or accuracy of the measurements, in the study by Adler et al., [10], on the one hand, the reliability was excellent for body volume (ICC = 0.998), with a mean difference of 0.1 L for the standard position and 0.2 L for both the relaxed and exhaled positions. On the other hand, reliability was good for fat mass percentage (ICC = 0.982), with a mean difference of −0.4%, 0.2% and 0.3% for the standard, relaxed and exhaled positions, respectively. Excellent accuracy was also found for body volumes (ICC = 0.952–0.999), with a root-mean-square coefficient of variation (RMS-%CV) of 1.9 to 2.3% (average three 3D scans); furthermore, higher accuracy was observed for total body volume (RMS-%CV < 1% for all), followed by torso volume (~1.2%), leg volume (~2.5%) and arm volume (3–5%) [20]. Finally, in the study by Ng et al. [14] 3D estimates were less accurate than DXA estimates, as 3D measurement accuracy metrics were generally one to three times the magnitude of the corresponding DXA accuracy metrics.

## 4. Discussion

The results found in this work showed that measurements made by different 3D scanners correlated highly with measurements made by other techniques, such as conventional anthropometry, ADP and DXA, among others. Although significant differences between methods were found in 12 studies, accuracy and/or reliability was higher for 3D scanners in seven of them [12,13,15,16,22,24,25]. Furthermore, in three other studies, the accuracy and/or reliability was excellent for 3D scanners [10,20,23]. On the other hand, in another article, the same strong correlations were observed between 3D and manual measurements, and in turn, no significant differences were observed between methods [17]. Finally, Vonk & Daanen [19] found similar results for one of the scanners analyzed; despite the significant differences between the six 3D and manual measurements, the correlations among three were high. For the other scanner, although only the differences for waist circumference reached significance, the correlations between the measurements obtained using both methods were weak.

Overall, the results of the studies reviewed suggest that 3D scans are a good method for assessing body composition, as they provide reproducible, accurate and reliable data that correlate strongly with those obtained by other techniques.

In this review, in most of the included studies (61.1%), 3D scanners were either more accurate than the other measurement techniques used, or these 3D scanners had excellent accuracy or reliability. These results are consistent with those obtained in other studies. For example, in the study by Wang et al. [26], the reliability for the 3D scanner was high, with an ICC greater than 0.99 and a CV less than 0.9% for chest, waist, hip, thigh and knee circumferences. Similar results have been found in previous validation studies, with higher accuracy and repeatability rates for measurements performed by 3D scanners [27,28,29]. Jaeschke et al. [29], on the other hand, found that measurements taken by 3D scanner were greater than manual measurements, although both were strongly correlated (men: waist circumference, mean difference (d) = 1.5 cm; r = 0.97 and hip circumference, d = 2.3 cm; r = 0.97; women: waist circumference, d = 4.7 cm; r = 0.96 and hip circumference, d = 3.0 cm; r = 0.98). Furthermore, they stated that the reliability was high for all 3D measurements (ICC > 0.98); these findings coincide with the results obtained in most of the studies included in this review.

The use of accurate body volume meters is a future challenge because three-dimensional scanners are faster, less expensive, less cumbersome and less invasive than are traditional body assessment methods [3,4,6]. Three-dimensional scanners could play an important role in nutrition and dietetics clinics. Kuzmar et al. [30] stated that incorporating techniques based on the use of 2D images enhances the cognitive experience of patients in dietary treatment. It is to be expected then that these 3D scanners, in addition to achieving an automated body assessment, faster and simpler than other body assessment techniques, would allow subjects, thanks to the creation of avatars (computerized representation of oneself), to improve their cognitive experience in the weight loss process. This possible improvement, in turn, could lead to stronger adherence. Moreover, future research should evaluate individuals with higher BMI since in some studies precision of the scanners decreased as the body mass index increased [13,23]. This decrease in precision is largely attributed to inconsistencies in landmarking and partition positioning in the 3D surface scan analysis algorithms [18].

In other fields, such as medicine, 3D photography is also increasingly accepted as a useful clinical tool. Reports on facial plastic surgery, maxillofacial surgery, and breast surgery or reconstruction support 3D photography because of its ability to reliably and accurately detect changes in shape and volume in 3D [16].

The present systematic review has a number of limitations that require areas of future research. First, the search only included publications in English and Spanish; therefore, all evidence available to date may not be represented. Second, the data sources used for this review have been Pubmed, Medline and the Cochrane Library; in future studies searches could be expanded using databases such as Web of Science, CINAHL Complete, or Embase, among others. Third, the present work included studies with small sample sizes, which may have led to greater uncertainty regarding the measured effect. Finally, the fact that only healthy adult subjects were evaluated in most of the studies, in whom body shape is stable, does not allow knowing whether these methods are equally useful for people with conditions that affect body composition. In addition, it is important to evaluate children and adolescents by adapting these types of measurements to the anthropometric characteristics of the individuals. However, this study does have strengths; for example, it is one of the few systematic reviews that attempts to gather the existing information in the literature, in relation to the validation of different 3D scanners for taking anthropometrics.

## 5. Conclusions

Three-dimensional scanners appear to be a good technology for body assessments, allowing automated, quick and easy body measurements, such as circumferences, body volumes and fat mass, among others.

These 3D scanners seem to provide reproducible, accurate and reliable data that correlate well with the different techniques used for the evaluation of body measurements (conventional anthropometry, DXA, ADP, electrical bioimpedance, etc.). To further improve this measurement technique, future research should focus on assessing both individuals with conditions affecting body composition and individuals with a high BMI because in some studies, the accuracy of the scans decreased with increasing body mass index.

## Figures and Tables

**Table 1:** PubMed search strategy.

Search Strategy	
#1	(“whole body imaging [MeSH Terms] OR “body scanner” [Title/Abstract] OR “body scanning” [Title/Abstract] OR “3d images” [Title/Abstract] OR “three dimensional imaging” [Title/Abstract])
#2	(“anthropometry” [MeSH Terms] OR “anthropometrics” [Title/Abstract] OR “anthropometric measures” [Title/Abstract] OR “waist circumference” [MeSH Terms] OR “hip circumference” [Title/Abstract])
#3	(“reproducibility of results” [MeSH Terms] OR “validity” [Title/Abstract] OR “validation” [Title/Abstract] OR “reliability” [Title/Abstract])
#4	#1 AND #2 AND #3

**Table 2:** First ten questions from the AXIS tool.

Reference	1	2	3	4	5	6	7	8	9	10
Bragança et al., 2018 [4]	Yes	Yes	No	No	Don’t know/comment	Yes	Do not know/comment	Yes	-	No
Adler et al., 2017 [10]	Yes	Yes	No	Yes	Yes	Yes	Do not know/comment	Yes	-	Yes
Bourgeois et al., 2017 [11]	Yes	Yes	Yes	No	Do not know/comment	Yes	Do not know/comment	Yes	-	Yes
Medina-Inojosa et al., 2016 [12]	Yes	Yes	No	Yes	Yes	Yes	Do not know/comment	Yes	-	Yes
Ng et al., 2016 [13]	Yes	Yes	No	Yes	Yes	Yes	Do not know/comment	Yes	-	Yes
Ng et al., 2019 [14]	Yes	Yes	No	Yes	Yes	Yes	Do not know/comment	Yes	-	Yes
Brooke-Wavell et al., 1994 [15]	Yes	Yes	No	No	Do not know/comment	Do not know/comment	Do not know/comment	Yes	-	Yes
Weiss et al., 2009 [16]	Yes	Yes	No	No	Do not know/comment	Do not know/comment	Do not know/comment	Do not know/comment	-	No
Pepper et al., 2010 [17]	Yes	Yes	No	Yes	Yes	Yes	Do not know/comment	Yes	-	Yes
Harbin et al., 2017 [18]	Yes	Yes	No	Yes	Yes	Yes	Do not know/comment	Yes	-	No
Bragança et al., 2017 [3]	Yes	Yes	No	Yes	Do not know/comment	Yes	Do not know/comment	Yes	-	Yes
Vonk & Daanen, 2015 [19]	Yes	Yes	No	No	Do not know/comment	Do not know/comment	Do not know/comment	Do not know/comment	-	No
Tinsley et al., 2019 [20]	Yes	Yes	No	Yes	Yes	Do not know/comment	Do not know/comment	Yes	-	Yes
Ladouceur et al., 2017 [21]	Yes	Yes	No	No	Do not know/comment	Do not know/comment	Do not know/comment	Yes	-	Yes
Ramos-Jiménez et al., 2018 [22]	Yes	Yes	No	Yes	Yes	Do not know/comment	Do not know/comment	Yes	-	Yes
Kuehnapfel et al., 2016 [23]	Yes	Yes	No	No	Do not know/comment	Do not know/comment	Do not know/comment	Yes	-	Yes
Koepke et al., 2017 [24]	Yes	Yes	No	Yes	Yes	Yes	Do not know/comment	Yes	-	Yes
Lu & Wang et al., 2010 [25]	Yes	Yes	No	No	Do not know/comment	Do not know/comment	Do not know/comment	Yes	-	No

**Table 3:** Last ten questions from the AXIS tool.

Reference	11	12	13	14	15	16	17	18	19	20
Bragança et al., 2018 [4]	Yes	Yes	Do not know/comment	-	Yes	Yes	Yes	Yes	No	Yes
Adler et al., 2017 [10]	Yes	Yes	No	-	Yes	Yes	Yes	Yes	Yes	Yes
Bourgeois et al., 2017 [11]	Yes	Yes	Do not know/comment	-	Yes	Yes	Yes	No	No	Yes
Medina-Inojosa et al., 2016 [12]	Yes	Yes	Do not know/comment	-	Yes	Yes	Yes	No	Yes	Do not know/comment
Ng et al., 2016 [13]	Yes	Yes	Do not know/comment	-	Yes	Yes	Yes	Yes	No	Yes
Ng et al., 2019 [14]	Yes	Yes	Do not know/comment	-	Yes	Yes	Yes	Yes	No	Yes
Brooke-Wavell et al., 1994 [15]	No	No	Do not know/comment	-	Yes	Yes	Yes	No	Yes	Yes
Weiss et al., 2009 [16]	No	No	Do not know/comment	-	Yes	Do not know/comment	Yes	No	No	Yes
Pepper et al., 2010 [17]	Yes	Yes	Do not know/comment	-	Yes	Yes	Yes	Yes	No	Yes
Harbin et al., 2017 [18]	Yes	Yes	Do not know/comment	-	Yes	Yes	Yes	Yes	No	Yes
Bragança et al., 2017 [3]	Yes	No	Do not know/comment	-	Yes	Yes	Yes	No	No	Do not know/comment
Vonk & Daanen et al., 2015 [19]	Yes	Yes	Do not know/comment	-	Yes	Yes	Yes	Yes	No	Yes
Tinsley et al., 2019 [20]	Yes	No	Do not know/comment	-	Yes	Yes	Yes	Yes	No	Yes
Ladouceur et al., 2017 [21]	No	Yes	Do not know/comment	-	Yes	Do not know/comment	Yes	No	No	Do not know/comment
Ramos-Jiménez et al., 2018 [22]	Yes	No	Do not know/comment	-	Yes	Yes	Yes	Yes	No	Yes
Kuehnapfel et al., 2016 [23]	Yes	Yes	Do not know/comment	-	Yes	Yes	Yes	Yes	Yes	Yes
Koepke et al., 2017 [24]	Yes	No	Do not know/comment	-	Yes	Yes	Yes	Yes	Yes	Yes
Lu & Wang et al., 2010 [25]	Yes	Yes	Do not know/comment	-	Yes	Yes	Yes	No	Do not know/comment	Do not know/comment

**Table 4:** Description of the studies included in the review.

Reference	Country	Year	Average Age	BMI (kg/m^2^)	Sample No. (n)	Objective	Type of Study
Bragança et al., 2018 [4]	United Kingdom	2018	24.03	22.62	37 (17 F ^1^/20 M ^2^)	To compare two anthropometric data collection techniques, i.e., manual methods and a Kinect-based 3D body scanner, to understand which provides more accurate and reliable results.	Cross-sectional study
Adler et al., 2017 [10]	Germany	2017	18–79	26.29	37 (17 F/20 M)	To investigate the longer-term validity and reliability of 3DPS-based body volume and %body fat over a period of approximately four weeks for application in epidemiological studies in the general adult population.	Cross-sectional study
Bourgeois et al., 2017 [11]	USA	2017	44	27.25	113 (73 F/40 M)	Critically evaluate three of these newer optical devices that differ in image acquisition and data processing technology, comparing body size and shape results with those obtained by reference methods.	Cross-sectional study
Medina-Inojosa et al., 2016 [12]	USA	2016	41.9	25.9	83 (40 F/43 M)	To evaluate the reliability and reproducibility of a 3D scanner in the measurement of anthropometric parameters in central obesity.	Cross-sectional study
Ng et al., 2016 [13]	USA	2016	44.45	26.4	37 (19 F/18 M)	Validate direct and derived anthropometrics of body composition from 3D scans of the whole body surface against criterion methods.	Cross-sectional study
Ng et al., 2019 [14]	USA	2019	44.8	27.2	407 (230 F/177 M)	Quantify the test-retest accuracy of 3DO PCA (principal component analysis) body composition estimates compared to DXA.	Cross-sectional study
Brooke-Wavell et al., 1994 [15]	United Kingdom	2009	27.9	-^3^	10 (5 F/5 M)	Compare the reliability and repeatability of LASS scanner and anthropometrics.	Cross-sectional study
Weiss et al., 2009 [16]	USA	2009	42.93	-	30 (28 F/2 M)	Compare the accuracy and reproducibility of manual measurements vs. 3D photographic measurements of the abdomen and thigh circumference.	Cross-sectional study
Pepper et al., 2010 [17]	USA	2010	29.64	25.57	70 F	Evaluate the reliability and validity of a 3D laser body scanner for estimating waist and hip circumferences and the waist-to-hip ratio.	Cross-sectional study
Harbin et al., 2017 [18]	USA	2017	22.1	24.5	265 (146 F/119 M)	Compare and validate the accuracy of a 3D infrared body scanner for determining body composition against hydrostatic weighing (HW), bioelectrical impedance analysis (BIA) and anthropometry (skinfold thickness and circumferences).	Cross-sectional study
Bragança et al., 2017 [3]	USA	2017	24.03	22.6	37 (17 F/20 M)	Compare anthropometric data collected using a Kinect body imaging system with data collected using traditional manual methods.	Cross-sectional study
Vonk & Daanen, 2015 [19]	Netherlands	2015	21.5	21.43	156 (27 F/219 M)	Evaluate the repeatability and validity of the SizeStream scanner and Poikos modeling system by scanning a large number of subjects multiple times.	Cross-sectional study
Tinsley et al., 2019 [20]	USA	2019	33.6	25.1	179 (103 F/76 M)	Quantify the test-retest accuracy (reproducibility) of four commercially available 3DO scanners for anthropometrics and examine the validity of total and regional body volume estimates produced by these scanners compared to reference methods.	Cross-sectional study
Ladouceur et al., 2017 [21]	Canada	2017	-	-	20 (9 F/11 M)	Develop a systematic method to compare manual and digital anthropometrics and validate a commercial 3D laser scanner for anthropometric measurements.	Cross-sectional study
Ramos-Jiménez et al., 2018 [22]	Mexico	2018	21.7	24.86	285 (140 F/145 M)	Validate a 3D image digitizer (TC2-18) to determine body dimensions in a fast and reliable manner.	Cross-sectional study
Kuehnapfel et al., 2016 [23]	Germany	2016	-	-	108 (69 F/39 M)	Compare 3D laser-based body scanners with classical manual anthropometrics (CA) with respect to feasibility, reliability and validity.	Cross-sectional study
Koepke et al., 2017 [24]	Switzerland	2017	24.55	22.97	123 M	Compare scanning and manual anthropometrics techniques based on five selected body measurements.	Cross-sectional study
Lu & Wang et al., 2010 [25]	China	2010	-	-	263 (91 F/172 M)	To evaluate scanned measurements in terms of accuracy and precision.	Cross-sectional study

^1^ Female. ^2^ Male. (In reference to the sex of the participants). ^3^ Information not reported in the paper.

**Table 5:** Statistical analysis, results and conclusions of the included articles.

Reference	Statistical Analysis	Results	Conclusions
Bragança et al., 2018 [4]	Accuracy: technical error of measurement (TEM) and relative technical error of measurement (%TEM). Reliability: Relative: interclass correlation coefficient (ICC) and reliability coefficient (R). Absolute: standard error of measurement (SEM) and coefficient of variation (CV).	Accuracy: TEM values < 2 cm. Higher manual technical accuracy (slightly lower values). %TEM: Only chest length obtained a value > 1.5% using the manual technique, while seven measurements did so using the 3D technique. Reliability: Relative (manual: ICC 0.80–0.99 and 3D: ICC 0.91–0.99). When comparing both methods, all the measurements, except neck circumference, presented slightly higher values using the manual technique. Very similar results for R (R > 0.95). Absolute: 3D technique results less reliable (higher SEM values), except neck circumference. According to CV, none of the methods performs well because for all measurements, the results were >5%.	Despite being considered sufficiently accurate and reliable for certain applications, the 3D scanner showed, for almost all measurements, a different result than obtained using the manual technique.
Adler et al., 2017 [10]	Validity: Pearson correlation coefficient and Bland-Altman plots. Q-Q plots to examine differences between 3D and ADP for body volume. Reliability: differences in 3D measurements, calculated as scan1 and scan2 and ICC.	Validity: 3D body volume and ADP strongly correlated (R = 0.99). ADP body volume 72.2 L and 3D body volume higher by 1.1 L (*p* < 0.001), 1.0 L (*p* < 0.001) and 2.5 L (*p* < 0.001) in standard, relaxed and exhaled positions, respectively. %MG 3D and ADP well correlated (R = 0.79), %MG ADP 23.75 and %MG 3D higher by 7.0% (*p* < 0.001), 6.6% (*p* < 0.001) and 16.6% (*p* < 0.001) for standard, relaxed and exhaled positions. Reliability: high for body volume, with a mean difference of 0.1 L, 0.2 L and 0.2 L for standard, relaxed and exhaled positions, respectively, and ICC > 0.98. %MG, standard position, mean difference of -0.4%, relaxed position 0.2%, and exhaled 0.3%, with ICCs of 0.982, 0.983 and 0.945, respectively.	Good agreement between 3D and ADP. Good validity and excellent reliability of the 3D scan.
Bourgeois et al., 2017 [11]	Comparison of measurements between methods: paired t-tests. Associations between methods: linear regression analysis. Bland-Altman plots.	Hip circumference: significant difference between conventional anthropometry and 3D scan (#1 and #2) (*p* < 0.0001). Waist: significant difference between conventional anthropometry and 3D scan (#2 and #3) (*p* < 0.0001). Arm: significant difference between conventional anthropometry and 3D scan (#1 and #3) (*p* < 0.0001). Thigh: significant difference between conventional anthropometry and 3D scan (#1 and 2) (*p* < 0.0001). Significant correlations between methods (R = 0.71–0.96; *p* < 0.0001 for all). Total body volume: significant difference between ADP and 3D measurements (*p* < 0.0001). Body volume measured by the three 3D scans were highly correlated with ADP volumes (R = 0.99 for all). Significant bias (*p* < 0.05) of −3.4 L, −2.4 L and −9.1 L for 3D scan (#1), (#2) and (#3), respectively; 3D systems underestimate body volume.	Reproducible measurements correlate well with reference methods.
Medina-Inojosa et al., 2016 [12]	Reproducibility: intraobserver and interobserver variability and paired *t*-test. Comparison between methods: unpaired t-test. ICC and Bland-Altman.	Intraobserver variations (reproducibility): 3.1 cm waist and 1.8 cm hip. Interobserver variations (precision): 3.9 cm waist and 2.4 cm hip. 3D scanner variability: 1.3 cm waist and 0.8 cm hip. Significant difference between methods (*p* < 0.05). ICC > 0.95 for all.	A 3D scanner is a more reliable and reproducible way to measure waist and hip circumference.
Ng et al., 2016 [13]	Agreement between methods: univariate linear regressions. Measurement biases between methods: Student *t*-test. % CV RMSE for paired test-retest measurements of the 3D scanner. R^2^.	Strong associations between methods for waist and hip circumference (R = 0.95 and 0.92, respectively). Significant differences of 1.75 cm for waist and 3.17 cm for hip between 3D and conventional anthropometry. Strong associations between 3D scan and ADP and DXA for total body volume (R = 0.99 and 0.97, respectively), with significantly lower volume measured by 3D scan compared to ADP (−4.15 L).	This study supports the use of 3D scanning as an accurate, reliable and automated surrogate for other methods.
Ng et al., 2019 [14]	Model accuracy/precision: R2 and RMSE. Measurement precision: RMSE and CV (%).	Precision of body composition comparing 3D scanner was DXA was as follows: fat mass, R = 0.88 male, 0.93 female; visceral fat mass, R = 0.67 male, 0.75 female. The test precision (test-retest) of the 3D scan for body fat was as follows: mean square error = 0.81 kg male, 0.66 kg female. Visceral fat according to 3D scan was as accurate (% CV = 7.4 for males, 6.8 for females) as that obtained using DXA (% CV = 6.8 for males, 7.4 for females).	The 3D estimates may be somewhat less accurate than DXA estimates.
Brooke-Wavell et al., 1994 [15]	Intraobserver and interobserver variability: standard error of measurement. Means, standard error of the mean and *t*-tests.	Comparison between methods (reliability): Women: significant differences (*p* < 0.05) between conventional anthropometry and 3D scan for neck, chest, waist width, waist depth and waist height. Men: significant difference between conventional anthropometry and 3D scan for neck circumference, chest and waist depth. Good agreement between methods (r = 0.964–0.998). Intraobserver Diff: 7 mm (larger) for waist circumference (manual). Only neck circumference, larger for 3D scan (5.3 mm vs. 3.0 mm). Interobserver difference (accuracy): 3.0–13.1 mm manual and 1.3–8.5 mm 3D scan.	3D measurements and anthropometry were generally similar. Larger interobserver differences for manual technique, lower precision.
Weiss et al., 2009 [16]	- ^1^	Intraobserver variations (reproducibility): researcher 1: 0.37 cm between repetitions, researcher 2: 0.406 cm and 3D: 0.171 cm. Very high correlations (r > 0.99), although higher 3D scan correlations (researcher 1 and 2 = 0.995 vs. 3D = 0.9988). Interobserver variations (precision): thigh circumference, variance 20.5% higher than variance for 3D scan. Abdominal circumference, variance 231.3% greater than the variance for the 3D scan.	Greater precision and reproducibility of the measurement with the use of the 3D scanner.
Pepper et al., 2010 [17]	Reproducibility: ICC and CV. Paired *t*-tests, correlation coefficients and Bland-Altman plots.	ICC > 0.99 for all circumferences measured by 3D. CVs showed little difference between intraindividual measurements, showing high agreement between repeated measurements (CV 0.53%-1.68%). No significant difference between methods for waist and hip (3D: 87.87 cm and 104.15 cm vs. conventional anthropometry: 87.73 cm and 104.39 cm, respectively *p* > 0.05). Highly correlated measurements (waist: r = 0.998 and hip: r = 0.984; *p* < 0.01).	3D scanner reliable and valid technique compared to conventional anthropometry.
Harbin et al., 2017 [18]	Level of agreement between methods: Bland-Altman graphs. Mean differences in %MG estimation: multivariate ANOVA.	Significant difference (*p* < 0.001) between %MG measured by 3D scan and the other methods (3D %MG: 18.1%; hydrostatic weighing %MG: 22.8%; bioelectrical impedance %MG: 20.1%; folds %MG: 19.7%; circumferences %MG: 21.2%). Bonferroni post hoc analysis revealed that the %MG estimated by 3D scan was significantly lower than that estimated by all other techniques.	Advances must be made before 3D scans can be designated as an accurate method.
Bragança et al., 2017 [3]	Comparison between methods: paired *t*-test.	Significant difference between various 3D measurements and conventional anthropometry (*p* < 0.001): shoulder width, back length, waist circumference, hip circumference, thigh circumference, knee circumference and ankle circumference.	Reliability and accuracy depend on the ability to remain static.
Vonk & Daanen, 2015 [19]	Repeatability: (ICC, ICC < 0.80: measurements with low repeatability. Accuracy: SEM, SEM > 10mm: not accurate enough. Validity: paired *t*-tests.	SizeStream scan: 120 measurements: ICC > 0.90 and 20 measurements: ICC < 0.80. Mean SEM: 10.1 mm. Validity: 6 measurements by 3D and conventional anthropometry: significant difference (*p* < 0.001) (chest, waist, hip, wrist, neck-bust distance and arm length). However, strong and significant correlations for chest (r^2^= 0.95; *p* < 0.001), waist (r^2^ = 0.92; *p* < 0.001) and hip (r^2^ = 0.96; *p* < 0.001). Poikos scanner: 14 measurements: ICC < 0.80 and 2 measurements: ICC > 0.90. Mean SEM: 54.5 mm. Significant difference only for waist (*p* < 0.001), but weak correlations (R^2^ < 0.60).	Only three of the six measurements compared could be validated (SizeStream scanner). Poikos is promising but less repeatable and valid than the SizeStream scanner.
Tinsley et al., 2019 [20]	Accuracy: ICC and RMS-%CV. Validity (regional and total volumes only): one-way ANOVA. Coefficient of determination (R^2^). RMSE. Bland-Altman with linear regression to evaluate the degree of proportional bias.	Accuracy: circumferences (ICC from 0.974 to 0.999) and volumes (ICC from 0.952 to 0.999). Average of four scans for RMS-%CV: circumferences (1.1% to 1.3%) and body volume (1.9% to 2.3%). Circumference highest accuracy: hip (RMS-%CV < 1% for all), waist (0.7–1.6%), thigh (0.8–1.4%) and arm (1.4–2.8%). Volume highest accuracy: total (RMS-%CV < 1% for all), torso volume (approx. 1.2%), leg (approx. 2.5%) and arm (3–5%). Validity: very strong linear relationships between methods for total body volume (R: 0.98–1.0), but SizeStream significantly overestimated it, and Styku underestimated it. Stronger relationship between 3D and DXA for torso volume (R: 0.96–0.97) than arm and leg volume (R: 0.65–0.93). However, all 3D scans significantly overestimated torso volume and underestimated arm and leg volume.	Excellent accuracy; however, relatively poor validity for total and regional body volume.
Ladouceur et al., 2017 [21]	Concurrent validity between methods: Pearson product moment correlation coefficient (PPMC) and paired t-test. Systematic error between the two methods: paired t-test. Bland-Altman.	Significant difference between conventional anthropometry and 3D measurements (*p* = 0.000).	The results of this study have shown promise for the future.
Ramos-Jiménez et al., 2018 [22]	Differences between methods: *t*-test for independent samples. Significance of finding differences, was analyzed using Cohen’s d. Linear regression for strength of associations.	3D measurements highly correlated with those of conventional anthropometry and plestimography. (R ≥ 0.75) but significantly different for all (*p* < 0.01).	Valid and reliable measurements when evaluating adult individuals; however, it is important to minimize body motion.
Kuehnapfel et al., 2016 [23]	Concordance of paired measurements: overall concordance correlation coefficient (OCCC). Illustration of results: scatter and Bland-Altman plots.	Validity: excellent for height (OCCC = 0.995), weight (OCCC = 1.00), waist (OCCC = 0.982), hip (OCCC = 0.938) and calf (OCCC = 0.988); good for arm (OCCC = 0.720); moderate for thigh (OCCC = 0.557). Notable bias between anthropometry and 3D measurements.	Reliability of 3D measurements was generally excellent or good, with some exceptions.
Koepke et al., 2017 [24]	Repeatability and agreement between repeated measurements within each method: mean differences, ICC, precision, and paired *t*-tests. Agreement between methods: mean differences (mSM, mMM), correlation coefficients, and paired *t*-tests. In addition, Lin’s coefficient of concordance.	3D: no significant difference between repeated measurements and strong correlations: chest: 0.981; *p* = 0.486; waist: 0.993; *p* = 0.397; buttocks: 0.997; *p* = 0.052; hip: 0.994; *p* = 0.280. Manual: chest: 0.968; *p* < 0.001; waist: 0.990; *p* < 0.001; buttocks: -0.955; *p* = 0.018; hip: 0.972; *p* = 0.186. Precision higher than 2.50 cm, up to 8.19 cm, indicating high disagreement. CCC remains high (>0.94) for height and waist. CCC = 0.781 for chest, 0.784 for hip and 0.258 for buttocks. However, significant difference between methods, (chest: +3.88cm *p* < 0.001); (waist: +1.17 cm *p* < 0.001); (buttocks: +12.62 *p* < 0.001); (hip: +4.37 *p* < 0.001).	Better accuracy and repeatability for 3D scanner. Highly correlated data, but important systematic differences. Therefore, the two techniques are not directly equivalent.
Lu & Wang., 2010 [25]	Paired t-test and MAD (mean absolute difference) between scan-derived measurement and manual measurement for each dimension as a measure of accuracy performance.	Accuracy: significant difference between methods for chest circumference (*p* = 0.0008) and waist circumference (*p* = 0.0090) but not hip circumference (*p* = 0.5974). Most MADs between scan-derived and manual measurements exceeded ISO 20685 criteria. Accuracy: MADs of all repeated measurements were less than 7 mm. When compared to the maximum allowable interobserver error reported in ANSUR, the accuracy of the 3D measurements was higher than that of the manual measurements.	3D measurements more accurate than manual measurements.

^1^ Information not reported in the article.

## Supplementary Materials

The following are available online at https://www.mdpi.com/article/10.3390/ijerph18126213/s1, Table S1: Data related to the measurement process.
