# Supplementary material for: Comparison of Body Scanner and Manual Anthropometric Measurements of Body Shape: A Systematic Review
Source: Int J Environ Res Public Health. 2021 Jun 8;18(12):6213. doi: 10.3390/ijerph18126213 (PMC8230172; doi:10.3390/ijerph18126213)
Supplement: Supplementary file 1 [file ijerph-18-06213-s001.zip › ijerph-1239001-supplementary.pdf]

Table S1. Data related to the measurement process.

Reference	Measurement techniques used	Person in charge of measuring	Clothing	Position	Number of times measured	Measures
Bragança et al., 2018 [4]	3D scanner and conventional anthropometry.	Certified anthropometrist	Underwear.	Standing stationary.	2 with each technique	Circumferences, widths and lengths.
Adler et al., 2017 [17]	3D scanner and ADP.	-	Underwear and bathing cap.	-	3 DPS: 3 scanners in 3 different postures<br>ADP: 2	Body volume and %body fat.
Bourgeois et al., 2017 [18]	3D scanner, conventional anthropometry, DXA and ADP.	Trained personnel	Tight-fitting clothes.<br>DXA: underwear and examination gown.	-	Weight and size: 2 times<br>Circumferences: 3 times	Circumferences and total body volume.
Medina-Inojosa et al., 2016 [10]	3D scanner and conventional anthropometry.	Previously trained students	No shoes, tight gray underwear and bathing cap.	Anatomically immobile standing position at the end of expiration.	-	Weight, height and circumferences.
Ng et al., 2016 [11]	3D scanner, DXA, ADP and conventional anthropometry.	-	Tight-fitting underwear and bathing cap.<br>DXA: gown, no shoes.	-	1 with each technique	Circumferences and volumes.
Ng et al., 2019 [20]	3D scanner and DXA.	-	Tight-fitting clothes.	Standard position.	2 with each technique	Total body mass, total and regional body fat.
Brooke-Wavell et al., 1994 [9]	3D scanner and conventional anthropometry.	Two experienced observers	-	As upright and still as possible.	2 with each technique	Circumferences.
Weiss et al., 2009 [21]	3D scanner and conventional anthropometry.	Trained researchers	-	-	2 with each technique	Circumferences.
Pepper et al., 2010 [15]	Three-dimensional 3D scanner and conventional anthropometry.	-	Scanner: tight-fitting white underwear, eyes protected by a blindfold.	Legs and arms slightly abducted to minimize occlusion.	Anthropometry: 1 time<br>Scanner: 2 times	Weight, size and circumferences.

Harbin et al., 2017 [29]	3D scanner, hydrostatic weighing, electrical bioimpedance and conventional anthropometry.	-	-	3D scanner: upright with feet forward, arms at a 45-degree angle to the torso.	Hydrostatic weighing: 3 times	Body mass and %body fat.
Bragança et al., 2017 [3]	3D scanner and conventional anthropometry.	-	Tight-fitting clothes.	Contained breathing.	5 times each technique	Circumferences, widths and lengths.
Vonk & Daanen, 2015 [16]	3D scanner and conventional anthropometry.	Students	Underwear. Tight-fitting shorts allowed.	Standing, leg extended and elbows slightly bent.	2 with each scanner	Circumferences.
Tinsley et al., 2019 [19]	4 3D scanners, DXA and ADP.	-	3 DO scanners and ADP: compression shorts and bathing cap.<br>DXA: lightweight sportswear.	-	2 with each scanner	Circumferences and total body volume.
Ladouceur et al., 2017 [13]	3D scanner and conventional anthropometry.	-	Tight-fitting white underwear.	3D scanner: still and posture (measure height).	3 with each technique	Circumferences, widths and depth.
Ramos-Jiménez et al., 2018 [14]	3D scanner and conventional anthropometry.	Certified Anthropometrist	Tight-fitting swimsuit and cap.	-	-	Circumferences.
Kuehnapfel et al., 2016 [23]	3D scanner and conventional anthropometry.	-	Underwear and socks.	Upright and relaxed.	3 with each technique	Circumferences: arm, waist, hip, thigh, calf.
Koepke et al., 2017 [12]	3D scanner and conventional anthropometry.	Trained anthropometrists	Tight-fitting underwear (+3D cap).	Standing, relaxed.	2 with each technique	Circumferences.
Lu & Wang., 2010 [22]	3D scanner and conventional anthropometry.	Trained personnel	Scanning suit and a cap.	Standing and holding their breath.	3 with each technique	Circumferences.
